# Effects of Curcumin on Parameters of Myocardial Oxidative Stress and of Mitochondrial Glutathione Turnover in Reoxygenation after 60 Minutes of Hypoxia in Isolated Perfused Working Guinea Pig Hearts

**DOI:** 10.1155/2016/6173648

**Published:** 2016-01-21

**Authors:** Ermita I. Ibrahim Ilyas, Busjra M. Nur, Sonny P. Laksono, Anton Bahtiar, Ari Estuningtyas, Caecilia Vitasyana, Dede Kusmana, Frans D. Suyatna, Muhammad Kamil Tadjudin, Hans-Joachim Freisleben

**Affiliations:** ^1^Department of Physiology, Faculty of Medicine, University of Indonesia, Jakarta 10430, Indonesia; ^2^Department of Pharmacology and Therapeutics, Faculty of Medicine, University of Indonesia, Jakarta 10430, Indonesia; ^3^National Cardiovascular Center, Harapan Kita Hospital and Department of Cardiology and Vascular Medicine, University of Indonesia, Jakarta 10430, Indonesia; ^4^Department of Medical Biology, Faculty of Medicine, University of Indonesia, Jakarta 10430, Indonesia; ^5^Medical Research Unit, Faculty of Medicine, University of Indonesia, Jakarta 10430, Indonesia

## Abstract

In cardiovascular surgery ischemia-reperfusion injury is a challenging problem, which needs medical intervention. We investigated the effects of curcumin on cardiac, myocardial, and mitochondrial parameters in perfused isolated working Guinea pig hearts. After preliminary experiments to establish the model, normoxia was set at 30 minutes, hypoxia was set at 60, and subsequent reoxygenation was set at 30 minutes. Curcumin was applied in the perfusion buffer at 0.25 and 0.5 *μ*M concentrations. Cardiac parameters measured were afterload, coronary and aortic flows, and systolic and diastolic pressure. In the myocardium histopathology and AST in the perfusate indicated cell damage after hypoxia and malondialdehyde (MDA) levels increased to 232.5% of controls during reoxygenation. Curcumin protected partially against reoxygenation injury without statistically significant differences between the two dosages. Mitochondrial MDA was also increased in reoxygenation (165% of controls), whereas glutathione was diminished (35.2%) as well as glutathione reductase (29.3%), which was significantly increased again to 62.0% by 0.05 *μ*M curcumin. Glutathione peroxidase (GPx) was strongly increased in hypoxia and even more in reoxygenation (255% of controls). Curcumin partly counteracted this increase and attenuated GPx activity independently in hypoxia and in reoxygenation, 0.25 *μ*M concentration to 150% and 0.5 *μ*M concentration to 200% of normoxic activity.

## 1. Introduction

Ischemia-reperfusion injury causes challenging problems in cardiovascular surgery [1–3]. Hypoxia and reoxygenation as a model for ischemia and reperfusion [4] have mainly been studied in isolated perfused rat hearts. Neely et al. [5] modified the original Langendorff preparation [6] into the working rat heart model, which we used to investigate the phenomenon of ischemia-reperfusion injury [7]. Now, we changed the rat heart model into isolated perfused working Guinea pig hearts.

Many chemicals used for cardio protection exert severe unwanted side effects. For this reason, natural and more moderate compounds are screened for their protective efficacy against ischemia-reperfusion injury [8]. Curcumin may be a suitable candidate [9] because it has a wide range of clinical activities [10] and structural similarities to antioxidants, for example, tocopherols and cardio protective compounds like flavonoids.

We present a first set of data about cardio protective effects of curcumin using the perfused isolated working Guinea pig heart model. The aims of this study were to find the appropriate experimental conditions for our model and to investigate effects of curcumin under these experimental conditions. In the future, we intend to use this model for the screening of extracts from Indonesian medicinal plants and to study their effects on hypoxia/ischemia-reoxygenation/reperfusion injury.

## 2. Materials and Methods

The complete isolated working heart perfusion device was established and all experiments were conducted at the Department of Physiology, Faculty of Medicine, Universitas Indonesia. Young adult Guinea pigs, aged one year and weighing 300–400 g, were obtained from the Animal Breeding Department of the Agricultural University of Bogor, Indonesia. All substances used (if not indicated differently) were purchased from Merck, Darmstadt, Germany, via their Indonesian subsidiary in Jakarta or from Sigma-Aldrich at highest purity available.

### 2.1. The Model

The arrangement of the perfusion apparatus had been described and schematically depicted by Deisinger and Freisleben [11]. Each Guinea pig was neck-fractured by rapid cervical dislocation, the heart was excised, and the aorta immediately connected to the aortic cannula of the apparatus and was perfused retrogradely as known from the Langendorff preparation. Subsequently, the perfusion cannula was connected anterogradely to the left atrium for normoxic perfusion with Krebs-Henseleit buffer [12] gassed with carbogen (O_2_ 95%/CO_2_ 5%). During hypoxia, hearts were perfused with the same buffer gassed with N_2_ 95%/CO_2_ 5%. In reoxygenation, conditions were readjusted to “normoxia,” that is, anterograde perfusion with carbogen-gassed buffer [7]. Normoxia and reoxygenation lasted 30 min, each, whereas hypoxic perfusion varied from 15 to 60 min.

For perfusion of the myocardium, it is important that the cannulation of the aorta does not disturb the access to the coronary arteries. The perfusate flows back to the right atrium mainly via the* sinus coronarius* and drops from the heart via the open pulmonary artery and* venae cavae* into a glass vessel, from where it flows back into the buffer circulation. Coronary flow was measured by the volume of perfusate that dropped per minute from the heart into a graduated glass vessel.

**Figure 1:**
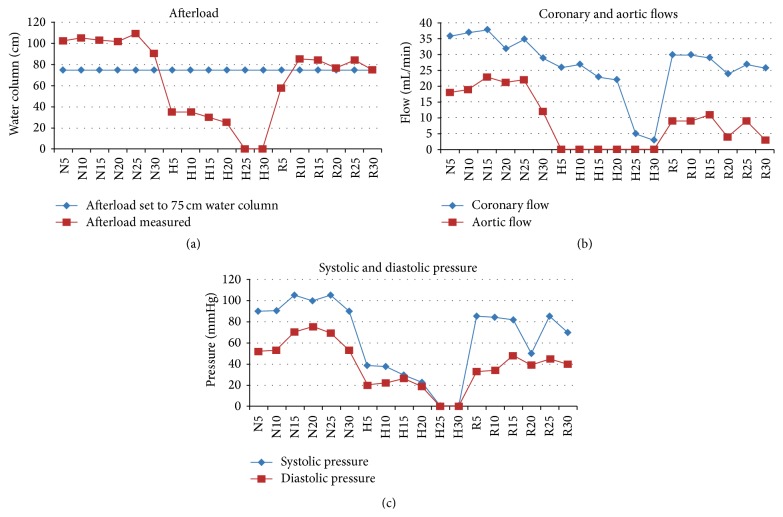
Parameters measured in preliminary experiments: (a) Afterload measured (red squares) during the course of 30 min of normoxia (about 100 cm water column) immediately dropped within 5 min of hypoxia to about 35 cm water column and then slowly decreased to zero between 10 and 25 min of hypoxia. In reoxygenation, afterload immediately improved again to 60 cm water column within 5 min and recovered to around 80 cm water column between 10 and 30 min of reoxygenation (red squares). To set up the “working” heart model, afterload was constantly set to 75 cm water column (blue diamond line). In subsequent experiments, the heart muscle had to pump against this pressure in its cardiac output (systolic phase). (b) Due to our model, aortic flow (red squares) was zero during hypoxia; it was around 20 mL/min in normoxia and recovered to about 10 mL/min (about 50% of normoxia) during the first 15 min of reoxygenation and then started to fluctuate and slowly decreased to almost zero by the end of experimental reoxygenation (R30). Coronary flow (blue diamonds) slowly decreased during normoxia and hypoxia from 35–40 mL/min to 3–5 mL/min by the end of hypoxia and then recovered rapidly to 30 mL/min in the beginning of reoxygenation and subsequently decreased slightly to 25 mL/min within 30 min of reoxygenation. (c) Systolic pressure (blue diamonds) was 90–100 mmHg in normoxia, dropped to 40 mmHg within 5 min of hypoxia, and further decreased to zero in 25 min of hypoxia. Within 5 min of reoxygenation, systolic pressure immediately recovered to 80–85 mmHg for 15 min and then started to fluctuate between 50 and 85 mmHg until the end of the experimental reoxygenation (R30). Diastolic pressure (red squares) showed similar course at a lower level with normoxic pressure of 55–75 mmHg, decreased to 20–25 mmHg in hypoxia, and dropped to zero after 20–25 min of hypoxia. Recovery during reoxygenation was stable between 35 and 45 mmHg until the end of experimental reoxygenation. These data in preliminary experiments served as the basis of setting up our model in subsequent main experiments with 60 min of hypoxia.

Afterload was measured as the height (cm) of the buffer column, which could be maintained by the aortic pressure. Aortic flow was determined as the volume (mL) per minute at a fixed afterload of 75 cm buffer column (Figure 1).

To measure systolic and diastolic pressure and heart rate a Nikon Kohden Polygraph was connected. This device also recorded the electrocardiogram, that is, initial arrhythmias; however, further electrocardiographic records were not in an interpretable quality (not shown). Hence, cardiograms were only used to decide about inclusion-exclusion criteria.

### 2.2. Experimental Procedures: Preliminary Experiments

We conducted preliminary experiments in order to

- set up the model and the sequential course of normoxia, hypoxia, and reoxygenation with 15 or 30 min of normoxic perfusion, 15, 30, or 60 min of hypoxia, and subsequent reoxygenation up to 30 min. In these experiments the excised hearts were examined for suitability, that is, stability over the time course in this model,
- determine inclusion-exclusion criteria and find out sensitive parameters for measurements in this model. Afterload was first determined in preliminary experiments and then adjusted to 75 cm water column in the main experiments (Figure 1),
- find appropriate concentrations of curcumin in this model. We applied 0.25, 0.5, and 1 *μ*M concentrations, given into the perfusate at 15 min of normoxia. After a first set of experiments, application of 1 *μ*M concentration was discontinued because we did not see dose-dependent effects and lower concentrations (0.25 and 0.5 *μ*M) were even more effective in our model (not shown).

In our preliminary experiments hematoxylin-eosin staining and histopathological examination were conducted using Olympus light microscope at 1000x magnification and compared to aspartate aminotransferase (AST) measurements. For the latter Merck kit (Cat. number 14829) with solution A containing Tris HCl pH 7.8, L-aspartate, malate dehydrogenase, and lactate dehydrogenase and solution B containing 2-oxoglutarate and NADH was used according to the manufacturer's manual with slight modification. One mL of solution A was mixed with 0.25 mL of solution B and incubated for 30 min. To 1 mL of this mixture 0.2 mL of perfusate was added and the absorption measured at 340 nm in Shimadzu spectrophotometer at 1, 2, and 3 min. In our preliminary experiments, we had tested the volumes needed for measurement of the low AST concentrations in the perfusate. The units per liter perfusate were then correlated to the weight of heart tissue and expressed as U × L^−1^ × g^−1^ heart tissue.

In the main experiments, histopathological examination was not continued, because histopathology correlated well with AST measurements and myocardial tissue was rather needed for the isolation of mitochondria and all other experimental procedures.

For statistical evaluation one-way ANOVA was used (normal parametric distribution); for nonparametric evaluation* post hoc* Tukey and Wilcoxon-Mann-Whitney *U* tests were applied. Results are presented as mean values ± standard deviation (SD); statistical significance is set to *p* < 0.05. Furthermore, the means of all normoxic values are set to 100% and the results of hypoxia, reoxygenation, curcumin 0.25 *μ*M, and curcumin 0.5 *μ*M concentrations expressed as percentage.

### 2.3. Inclusion-Exclusion Criteria

Our animal housing and experimental procedures strictly followed the Helsinki and Tokyo regulations on animal studies in their actual versions. Guinea pigs were kept in small groups of 3–5 animals with* ad libitum* access to food and water, in an acclimatized room with a window and thus natural day-and-night light fluctuations. Inclusion criteria for the main experiments were young adult Guinea pigs, aged one year; body weight 300–400 g; heart rate of the isolated perfused heart between 150 and 250 beats per minute; and coronary flow of the isolated perfused heart during normoxia between 20 and 50 mL.

Exclusion criteria for the main experiments were time between excision of the heart and retrograde perfusion more than 3 min; initial arrhythmias after cannulation longer than 3 min; and afterload = aortic pressure performance: less than 75 cm water column in normoxia.

### 2.4. Main Experiments

Conditions in the main experiments are indicated where necessary. Protein was measured according to Lowry et al. [13]; thiobarbituric acid-reactive substances (TBARS) were determined according to Chirico [14] using Waters HPLC device with a Spherisorb 5ODS2-C18 column. TBARS are expressed as malondialdehyde (MDA) throughout the text. Glutathione (GSH) was determined with the method of Ellman [15].

Mitochondria were prepared as described by Mela and Seitz [16] omitting nagarse. Relative specific activity of succinate dehydrogenase (SDH) was measured for control: 40 *μ*L of the sample was added into a cuvette containing a mixture of 120 *μ*L NaP_*i*_ 800 mM, pH 7.6; 120 *μ*L KCN 10 mM; 240 *μ*L sodium succinate 100 mM; 48 *μ*L 2,6-dichlorophenolindophenol 1 mM; and 632 *μ*L distilled water. The absorption at *λ* = 600 nm was followed for 5 min at 37°C in a Shimadzu spectrophotometer equipped with a thermostat cuvette holder. We measured relative specific SDH activities between 7.89 and 12.72 to characterize our mitochondrial preparations [17].

Enzyme activity of glutathione peroxidase (GPx) and glutathione reductase (GR) were measured as described by Flohé and Günzler [18] with slight modifications: our reaction mixture contained 200 *μ*L GSH 20 mM; 200 *μ*L sodium azide 20 mM; 200 *μ*L EDTA 20 mM; 200 *μ*L NADPH 1 mM; and 100 *μ*L NaP_*i*_ 0.1 M, pH 7.0. For measurement of GPx activity, 20 *μ*L glutathione reductase 1 U was added and incubated with 60 *μ*L isolation buffer at 37°C for 10 min. Subsequently, 20 *μ*L of mitochondrial preparation was added and absorption followed for 5 min at *λ* = 340 nm. For measurement of glutathione reductase, instead of 20 *μ*L glutathione reductase 1 U, glutathione peroxidase was added, accordingly. All photometric measurements were accomplished in a Shimadzu spectrophotometer equipped with a thermostat cuvette holder.

## 3. Results

### 3.1. Preliminary Experiments

The time course of our model with normoxia, hypoxia, and reoxygenation, 30 min each, is depicted in Figure 1. In our preliminary experiments, hypoxia varied, 15, 30, or 60 min; throughout the main experiments hypoxia was set to 60 min.

Heart rate was between 173 and 217 beats per minute and turned out to be a stable parameter in our experimental setting: it recovered after 15 and 30 min of hypoxia in reoxygenation to 100.5% and 98.9%, respectively, without curcumin, and to 97.1% and 101.7%, respectively, with 0.25 and 0.5 *μ*M curcumin. Differences between all these values were statistically not significant indicating that the hearts were stable over the experimental course of our model.

For measurement of systolic pressure a water column in our device [7, 11] with normoxic values up to 100 cm was used corresponding to and exceeding the constant afterload water column of 75 cm, against which the isolated heart had to perform its work. In addition, systolic and diastolic pressure were measured using a mercury manometer (Figure 1(c)).

#### 3.1.1. Histopathology and Aspartate Aminotransferase (AST)

Histopathological changes depended on the duration of hypoxia: in hypoxia up to 15 min we did not observe changes in tissue ultrastructure; however, changes became visible at 30 min and, particularly after 60 min, destruction of the myocardium was obvious (not shown). This result mirrored the measurement of AST activity in the perfusate: with only after 60 min of hypoxia, AST activity increased significantly during reoxygenation indicating tissue damage and release of AST from cardiomyocytes into the perfusate.

### 3.2. Main Experiments

For the main experiments, the following experimental settings were chosen: normoxia 30 min, hypoxia 60 min, and subsequent reoxygenation 30 min. Curcumin was injected into the perfusion buffer at 0.25 *μ*M or 0.5 *μ*M concentrations after 15 min of normoxic perfusion.

#### 3.2.1. Cardiac Parameters

Cardiac parameters measured were systolic pressure, aortic flow, and coronary flow (Table 1).

#### 3.2.2. Myocardial Tissue

Myocardial tissue parameters measured are shown in Table 2.

*(1) Aspartate Aminotransferase (AST).* After 60 min of hypoxia, AST activity increased significantly during reoxygenation indicating tissue damage and release of AST from cardiomyocytes into the perfusate (Table 2). Curcumin, at 0.25 *μ*M concentration, protected the myocardium from AST release significantly from 139.9% in reoxygenation to normoxic values. This protective effect was significant with 0.25 *μ*M (*p* < 0.05) but not with 0.5 *μ*M concentration (Table 2).

*(2) Thiobarbituric Acid-Reactive Substances (TBARS)/Malondialdehyde (MDA).* Lipid peroxidation (LPO) is considered a major reason of membrane destruction; LPO is often determined via thiobarbiturate reaction and expressed through its byproduct malondialdehyde (MDA). As can be seen in Table 2, MDA increased to 232.5% of normoxia (8.33 ± 5.30 nmol × g^−1^ tissue, 100%) during reoxygenation (19.37 ± 8.31 nmol × g^−1^ tissue) after 60 min of hypoxia. Although during hypoxia 0.5 *μ*M concentration of curcumin had a stronger effect than 0.25 *μ*M, both concentrations equally reduced MDA levels to 150% in reoxygenation.

#### 3.2.3. Mitochondrial Parameters

The mitochondrial parameters measured are presented in Table 3.

*(1) Thiobarbituric Acid-Reactive Substances (TBARS).* Mitochondrial TBARS levels are expressed as nmol MDA per g mitochondrial protein (*n* = 6). We found higher levels in normoxic mitochondria than in myocardial tissue if correlated to mitochondrial protein, 17.85 ± 2.79 (100%). During hypoxia the increase to 119% was only moderate but significant in reoxygenation, 165.4% (*p* < 0.05 versus N). Curcumin diminished mitochondrial MDA levels to normoxia at 0.25 *μ*M concentration (R versus R_0.25_; *p* < 0.05), whereas 0.5 *μ*M concentration of curcumin was less effective (Table 3).

*(2) Reduced Glutathione (GSH).* Mitochondrial GSH decreased from 40.6 ± 10.9 nmol × mg^−1^ protein in normoxia (100%) to 56.4% in hypoxia (*p* = 0.011 versus N) and 35.2% in reoxygenation (*p* < 0.001 versus N). During hypoxia, curcumin did not have much effect, but in reoxygenation it significantly increased GSH at both concentrations (Table 3).

*(3) Glutathione Peroxidase (GPx).* Glutathione peroxidase activity was 324.1 ± 115.0 nmol × min^−1^ × mg^−1^ protein in normoxia (100%). During hypoxia GPx activity increased slightly to 121.1% and in reoxygenation significantly to 255.2%. Curcumin, at 0.25 *μ*M concentration, attenuated GPx activity to about 150% and at 0.5 *μ*M concentration to about 200%, both during hypoxia and reoxygenation (Table 3).

*(4) Glutathione Reductase (GR).* Glutathione reductase activity was 77.2 ± 15.7 nmol × min^−1^ × mg^−1^ protein in normoxia (100%). During hypoxia GR activity decreased moderately to 58.8% and in reoxygenation significantly to 29.3% (*p* < 0.05). During hypoxia, curcumin did not have a significant effect on GR activity, but, in reoxygenation, 0.25 *μ*M concentration increased GR activity to 46.8% and 0.5 *μ*M concentration significantly increased GR activity to 62.0% (R versus R_0.5_ *p* = 0.006). Interestingly, values with curcumin were almost the same during hypoxia and reoxygenation, 48.8% and 46.8% at 0.25 *μ*M concentration, 62.5% and 62.0% at 0.5 *μ*M concentration (Table 3).

## 4. Discussion

In our experiments Guinea pig hearts were more stable than rat hearts over the experimental time course of one hour and thus more suitable to our experimental conditions in Jakarta. The discussion about the stability of the performance of isolated perfused hearts goes back almost four decades; isolated perfused Guinea pig hearts had been considered more stable than rat hearts by some working groups, whereas others could not confirm any differences, which may strongly depend on the experimental conditions [19–26]. We do not want to go into the discussion about differences in metabolic pathways (e.g., endogenous synthesis of ascorbic acid) and whether environmental (e.g., tropical) conditions may play a role. However, we state from our own experience that the slightly bigger Guinea pig hearts make it easier to fix them to the cannulas of the apparatus and thus reduce initial complications such as arrhythmias.

### 4.1. Main Experiments: Cardiac Parameters

In the main experiments hypoxia was set to 60 min and systolic pressure, aortic flow, and coronary flow were measured as cardiac parameters. Two concentrations of curcumin in the reoxygenation buffer were applied, 0.25 *μ*M and 0.5 *μ*M, but, generally, 0.5 *μ*M curcumin did not exert higher effects than 0.25 *μ*M concentration (after we had already ruled out 1 mM concentration of curcumin in our preliminary experiments).

Systolic pressure recovered during reoxygenation by 55-56%, under 0.25 *μ*M curcumin between 85% and 90% and under 0.5 *μ*M curcumin between 83% and 88%. Aortic flow recovered to 31-32% during reoxygenation, under 0.25 *μ*M curcumin between 45% and 50% and under 0.5 *μ*M curcumin between 47% and 50%.

Coronary flow exerted an overshooting reaction of almost 170% during reoxygenation. This overshoot might depend on the experimental setting of the isolated heart. Coronary flow was measured as the amount of buffer dropping down from the heart's perfused coronary arteries and* sinus coronarius*. Hence, the “coronary flow” especially during reoxygenation might be a mixture of coronary perfusion and leakiness of coronary vessels. The increase of about 70% over the normoxic value was certainly due to the leakiness of the coronary blood vessels after 60 minutes of hypoxia.

Leakiness of blood vessels is a well-known and dreadful reoxygenation/reperfusion phenomenon often causing considerable edema in tissues as part of ischemia-reperfusion injury [1–3]. Hence, the influence of curcumin in lowering this overshoot can be considered a positive therapeutic effect. Curcumin, at 0.25 *μ*M concentration, reduced the overshoot to roughly 140% and at 0.5 *μ*M concentration to about 138%. Thus, coronary flow under the aspect of ischemia-reperfusion injury did not improve much better with 0.5 *μ*M than with 0.25 *μ*M concentration of curcumin.

### 4.2. Myocardial Tissue

After 60 min of hypoxia lipid peroxidation destroyed cell membranes and cardiomyocytes became leaky and released AST into the perfusate, where its enzymatic activity increased during reoxygenation; in parallel, also MDA increased to about 233%. During reoxygenation curcumin reduced AST activity significantly at 0.25 *μ*M (*p* < 0.05), but not at 0.5 *μ*M concentration (*p* > 0.05). Curcumin reduced MDA significantly during hypoxia only at 0.5 *μ*M, but not at 0.25 *μ*M concentration, whereas both concentrations reduced equally MDA tissue levels from 233% in reoxygenation to about 150%.

Thiobarbiturate is the mostly used reagent to measure lipid peroxidation (LPO) and the result is expressed as TBARS (thiobarbituric acid-reactive substances) or as one of the major byproducts of LPO, MDA. These parameters are widely used as markers of oxidative stress and tissue damage. Recently, discussions have been extended about the concept of oxidative damage and the value of LPO and MDA as generalized markers of oxidative stress in cells, tissues, and body fluids. We do not go into this discussion here; however, one point of criticism often raised is that the markers are not determined at the place of origin. Hence, we tried to overcome this point by differentiating MDA as a parameter of tissue damage, measured from myocardium, and MDA measured from mitochondria as a parameter of LPO in mitochondrial membranes.

In general, 0.5 *μ*M curcumin did not exert higher effects on myocardial tissue parameters (AST, MDA) than 0.25 *μ*M concentration.

### 4.3. Mitochondrial Parameters

Mitochondrial MDA level was only decreased significantly during reoxygenation by 0.25 *μ*M curcumin, but not by 0.5 *μ*M concentration. At both concentrations, curcumin increased significantly mitochondrial GSH content during reoxygenation, whereas, during hypoxia, no significant effects on MDA and glutathione were observed. We were interested in the activities of glutathione reductase and glutathione peroxidase under the same conditions: The activity of glutathione reductase mirrored well the content of reduced glutathione. During reoxygenation curcumin exerted a significantly stimulating effect on GR at 0.5 *μ*M, but not at 0.25 *μ*M concentration. At either concentration, GR activity was almost the same during hypoxia and reoxygenation.

Glutathione peroxidase was slightly increased in hypoxia, but during reoxygenation its activity was tremendously high, 255% of normoxic control values. Curcumin, at 0.25 *μ*M concentration, attenuated GPx to about 154% (*p* < 0.05) and at 0.5 *μ*M to 206% (*p* > 0.05). Interestingly, at both concentrations, GPx had the same activity in hypoxia and reoxygenation of about 150% (0.25 *μ*M curcumin) and 200% (0.5 *μ*M curcumin) of normoxic values. Hence, curcumin, in two concentrations used in our experiments, appeared to be an independent attenuator of both GR and GPx during hypoxia and reoxygenation. Interestingly, on GR 0.5 *μ*M curcumin had a stronger effect than 0.25 *μ*M concentration, whereas the overshoot of GPx activity in reoxygenation was downregulated more strongly by 0.25 than by 0.5 *μ*M of curcumin.

Protective effects through enhanced GPx during hypoxia and reoxygenation in other organs have been reported [27–29]; in human plasma GPx-3 isoenzyme was threefold increased over normal expression during hypoxia [30]. On the contrary, Manikandan et al. [31] reported decreased GPx activity during isoprenaline induced myocardial ischemia in rats. Curcumin at 15 mg × kg^−1^ body weight (pre- and postischemic application) managed to normalize GPx activity, whereas curcumin administered either pre- or postischemically had much lower effects.

Animal and organ distribution of cGPx was reported to be low in Guinea pig hearts [32] and less than one-third of the activity found in isolated mouse heart atria was detected in Guinea pigs [33]. On the other hand, overexpression of PHGPx protected a guinea pig cell line from LPO-mediated injury [34]. In mitochondria, two isoforms out of various GPx isoenzymes [35, 36] appear to exist, for example, cGPx (GPx-1) and PHGPx (GPx-4) [37, 38], and GPx-1 was reported to protect mouse heart mitochondria against hypoxia/reoxygenation injury [28]. Transgenic mice overexpressing cGPx were protected against cerebral ischemia/reperfusion injury and apoptosis induced by it, whereas cGPx-mutant mice and nontransgenic control animals were more susceptible [39]. In this context, selenium dependence of GPx isoenzymes should be considered, which differs “hierarchically” between isoforms [32] and we suggest that it should be the scope of future studies with our isolated perfused working guinea pig heart model [40].

During reoxygenation GPx was considerably increased to 255% of control values. Increased GPx activity consumes more GSH and produces GSSG; GR activity is needed to replenish reduced GSH, but diminished GR activity will lead to GSH depletion in hypoxia and reoxygenation. Attenuation of GPx activity by curcumin and concomitant slight stimulation of GR activity, as observed in our model, would help to prevent rapid GSH depletion.

At higher concentration up to 2.5 *μ*M curcumin was reported to protect rat liver mitochondria against* tert*-butylhydroxyperoxide- (*t*-BHP-) induced mitochondrial dysfunction, such as breakdown of transmembrane potential and swelling [17, 41]. On the other hand, at these concentrations curcumin was suspected to consume reduced glutathione [41]. It was assumed that curcumin participates in phenoxyl-radical recycling mechanisms as demonstrated for tocopherol and other phenolic compounds [42–44]. Such reductive “recycling” mechanisms involve reduced GSH [43, 44] and may consume glutathione at higher concentrations of curcumin (and other phenolic antioxidants) and thus contribute to GSH depletion during oxidative stress [17, 45]. Although these mechanisms still need further clarification, they may be the reason for prooxidant effects at higher concentrations; we had to rule out 1 *μ*M curcumin in our preliminary experimentation and did not see a clear advantage of 0.5 *μ*M over 0.25 *μ*M concentration in some results of our main experiments.

In literature, curcumin concentrations differ considerably between experimental settings. Hence, it is difficult to compare results, especially since pharmacokinetic data are often missing. Pan et al. [46] investigated pharmacokinetic parameters of curcumin in mice: one mg × kg^−1^ body weight peaked in 0.5 *μ*M plasma concentration, 50% of which was metabolized within 8 h. The concentrations in our perfusion buffer correlated to these data. In a Langendorff rat heart model [8] GPx was not much influenced by curcumin, whereas GR was stimulated. The differences with our results on GPx activity may be due to different animals and the experimental model. In the Langedorff preparation the isolated heart is perfused retrogradely, whereas in our model the isolated working heart is perfused anterogradely and its mechanical work is closer to physiological conditions. Their study, which only presented one value for ischemia-reperfusion (I/R) observed a decrease in GR activity during I/R to almost 47% [8]. Their value is very close to the average (about 44%) of our values in hypoxia (58.8%) and reoxygenation (29.3%). This decrease of GR during I/R can only be considered as an enzymatic or metabolic dysregulation, because the low GR activity cannot replenish higher GSH consumption by the oxidative stress during hypoxia/ischemia-reoxygenation/reperfusion, including the overshoot of GPx. Metabolic dysregulations (e.g., calcium paradox) have been observed in the scenario of ischemia-reperfusion injury [1–3].

Curcumin appeared to be an independent regulator of GR and GPx activities under our experimental conditions. In hypoxia and in reoxygenation, 0.25 *μ*M concentration of curcumin attenuated GPx activity to 150% and 0.5 *μ*M concentration of curcumin attenuated GPx to 200% of normoxic activity and, thus, moderately reduced the overshoot of GPx activity during reoxygenation. Concomitantly, curcumin attenuated GR during reoxygenation through moderate upregulation with 0.25 *μ*M curcumin and significant upregulation with 0.5 *μ*M concentration; in other words, towards GR 0.5 *μ*M curcumin was more effective than the lower concentration. This attenuating effect on GR activity by curcumin was also observed in the Langendorff preparation by the before-mentioned study [8].

These considerations demonstrate that the concentration of curcumin is crucial to its metabolic effects; that is, the dosage of curcumin determines whether it exerts therapeutic activity. Moreover, the therapeutic efficacy of curcumin appears to go beyond its antioxidant effects [47, 48]: curcumin exerts regulatory effects on expression and activity of the enzymes involved in mitochondrial glutathione turnover.

## 5. Conclusion

In our experimental setting of the isolated perfused working Guinea pig heart curcumin exerts protection against hypoxia-reoxygenation injury at 0.25 *μ*M and 0.5 *μ*M concentrations on cardiac parameters and myocardial tissue damage and on mitochondrial GSH turnover. The higher concentration did not exert advantages over the lower one on cardiac parameters and myocardial tissue. On parameters of mitochondrial GSH turnover measured in our study, 0.5 *μ*M concentration was only advantageous towards GR. In clinical cardio protective dose determination it should be considered that curcumin appears to have an upper dose limitation towards cardiac ischemia-reperfusion injury.

## Figures and Tables

**Table 1:** Cardiac parameters.

	Normoxia	Hypoxia 60 min	Reoxygenation
Systolic pressure: cm H_2_O (*n* = 9; mean ± SD)			
	94.61 ± 9.38	0	52.31 ± 6.12 (55.3%) *p* < 0.05
Curc. 0.25 *µ*M	92.73 ± 10.06	0	83.37 ± 7.96 (89.9%)
Curc. 0.5 *µ*M	95.12 ± 11.0	0	77.78 ± 10.24 (83.2%)
Normoxia (mean = 94.15 = 100%)			73.2%
Curc. 0.25 *µ*M			85.6%
Curc. 0.5 *µ*M			88.1%

Aortic flow: mL × min^−1^ (*n* = 9; mean ± SD)			
	14.13 ± 4.70	0	4.37 ± 3.85 (30.9%) *p* = 0.05
Curc. 0.25 *µ*M	12.20 ± 3.75	0	6.10 ± 3.34 (50.0%)
Curc. 0.5 *µ*M	14.27 ± 4.73	0	6.73 ± 4.33 (47.2%)
Normoxia (mean = 13.5 = 100%)			32.4%
Curc. 0.25 *µ*M			45.2%
Curc. 0.5 *µ*M			49.9%

Coronary flow: mL × min^−1^ (*n* = 9; mean ± SD)			
	9.53 ± 3.04	0	15.97 ± 7.39 (167.6%)
Curc. 0.25 *µ*M	10.53 ± 2.5	0	13.6 ± 3.56 (129.2%)
Curc. 0.5 *µ*M	8.9 ± 6.48	0	13.27 ± 7.19 (149.1%)
Normoxia (mean = 9.65 = 100%)			165.5%
Curc. 0.25 *µ*M			140.9%
Curc. 0.5 *µ*M			137.5%

**Table 2:** Myocardial tissue parameters.

	Normoxia	Hypoxia	Reoxygenation
Aspartate aminotransferase (AST)			
AST: U × L^−1^ × g^−1^ heart tissue (*n* = 5; mean ± SD)			
	2.934 ± 1.451 (100%)	3.249 ± 1.119 (110.7%)	4.105 ± 1.917 (139.9%)
			N versus R *p* = 0.05
Curc. 0.25 *µ*M		3.382 ± 2.149 (115.3%)	2.397 ± 0.901 (81.7%)
			R versus R_0.25_ *p* < 0.05
Curc. 0.5 *µ*M		3.671 ± 1.446 (125.1%)	3.192 ± 1.516 (108.8%)

TBARS, expressed as malondialdehyde (MDA)			
MDA: nmol × g^−1^ heart tissue (*n* = 6; mean ± SD; N versus R and H versus H_0.5_ *p* < 0.05)			
	8.33 ± 5.30 (100%)	9.36 ± 6.24 (112.4%)	19.37 ± 8.31 (232.5%; N versus R *p* < 0.05)
Curc. 0.25 *µ*M		8.86 ± 0.48 (106.4%)	12.46 ± 10.30 (149.6%)
Curc. 0.5 *µ*M		5.88 ± 5.85 (70.6%)	12.52 ± 6.85 (150.3%)
		H versus H_0.5_ (*p* < 0.05)	

The activity of AST was measured in the perfusate as U × L^−1^ and then correlated to the weight of the heart (= g heart tissue). TBARS, thiobarbituric acid reactive substances; N, normoxia; H, hypoxia; R, reoxygenation; R_0.25_, reoxygenation with curcumin (curc.) 0.25 *µ*M; H_0.5_, hypoxia with 0.5 *µ*M curc.

**Table 3:** Mitochondrial parameters.

	Normoxia	Hypoxia	Reoxygenation
MDA: nmol × mg^−1^ mitochondrial protein (*n* = 6; mean ± SD; N versus R and R versus R_0.25_ *p* < 0.05)			
	17.85 ± 2.79 (100%)	21.24 ± 3.53 (119.0%)	29.52 ± 9.47 (165.4%; N versus R; *p* < 0.05)
Curc. 0.25 *µ*M		17.68 ± 4.40 (99.1%)	16.47 ± 1.99 (92.3%; R versus R_0.25_; *p* < 0.05)
Curc. 0.5 *µ*M		19.93 ± 3.62 (111.7%)	24.45 ± 3.24 (137.0%)

GSH: nmol × mg^−1^ protein (*n* = 6 in each group; mean ± SD)			
	40.6 ± 10.9 (100%)	22.9 ± 10.7 (56.4%)	14.3 ± 4.3 (35.2%)
		N versus H (*p* = 0.011)	N versus R (*p* < 0.001)
Curc. 0.25 *µ*M		19.5 ± 7.1 (48.0%)	27.0 ± 11.2 (66.5%)
			R versus R_0.25_ (*p* < 0.05)
Curc. 0.5 *µ*M		23.2 ± 7.0 (57.1%)	28.3 ± 8.0 (69.7%)
			R versus R_0.5_ (*p* < 0.05)

GPx: nmol × min^−1^ × mg^−1^ protein (*n* = 6 in each group; mean ± SD)			
	324.1 ± 115.0 (100%)	392.2 ± 150.1 (121.1%)	826.1 ± 268.0 (255.2%)
		H versus R (*p* < 0.05)	N versus R (*p* < 0.05)
Curc. 0.25 *µ*M		513.3 ± 59.4 (158.6%)	498.6 ± 44.5 (153.8%)
			R versus R_0.25_ (*p* < 0.05)
Curc. 0.5 *µ*M		664.4 ± 206.3 (205.3%)	665.0 ± 219.1 (205.7%)

GR: nmol × min^−1^ × mg^−1^ protein (*n* = 6 in each group; mean ± SD)			
	77.2 ± 15.7 (100%)	45.3 ± 17.1 (58.8%)	22.2 ± 9.6 (29.3%; N versus R *p* < 0.05)
Curc. 0.25 *µ*M		36.9 ± 16.9 (48.8%)	35.4 ± 16.0 (46.8%)
Curc. 0.5 *µ*M		47.3 ± 18.6 (62.5%)	46.9 ± 18.5 (62.0%; R versus R_0.5_ *p* = 0.006)

MDA, malondialdehyde; GSH, reduced glutathione; GPx, glutathione peroxidase; GR, glutathione reductase; N, normoxia; H, hypoxia; R, reoxygenation; R_0.25_, reoxygenation with 0.25 *µ*M curcumin (curc.); R_0.5_, reoxygenation with 0.5 *µ*M curc.
